# Low temperature and low salinity drive putatively adaptive growth differences in populations of threespine stickleback

**DOI:** 10.1038/s41598-017-16919-9

**Published:** 2017-12-01

**Authors:** Taylor C. Gibbons, Seth M. Rudman, Patricia M. Schulte

**Affiliations:** Biodiversity Research Centre and Department of Zoology, 6270 University Blvd, University of British Columbia, Vancouver, BC V6T 1Z4 Canada

## Abstract

Colonisation can expose organisms to novel combinations of abiotic and biotic factors and drive adaptive divergence. Yet, studies investigating the interactive effects of multiple abiotic factors on the evolution of physiological traits remain rare. Here we examine the effects of low salinity, low temperature, and their interaction on the growth of three North American populations of threespine stickleback (*Gasterosteus aculeatus*). In north-temperate freshwater habitats, stickleback populations experience a combination of low salinity and low winter temperatures that are not experienced by the ancestral marine and anadromous populations. Here we show that both salinity and temperature, and their interaction, have stronger negative effects on marine and anadromous populations than a freshwater population. Freshwater stickleback showed only a ~20% reduction in specific growth rate when exposed to 4 °C, while marine and anadromous stickleback showed sharp declines (82% and 74% respectively) under these conditions. The modest decreases in growth in freshwater stickleback in fresh water in the cold strongly suggest that this population has the capacity for physiological compensation to offset the negative thermodynamic effects of low temperature on growth. These results are suggestive of adaptive evolution in response to the interactive effects of low salinity and low temperature during freshwater colonisation.

## Introduction

A growing number of studies have demonstrated that changes in abiotic factors can lead to local adaptation in physiological traits^1–6^. Yet most of these studies either do not address the specific abiotic factors driving divergence (i.e. they look for adaptation between environments that differ in many factors) or consider only a single abiotic factor in isolation. In natural environments, multiple abiotic factors can co-vary between localities and divergent natural selection is likely the result of responses to differences in multiple factors between environments^7^. Thus, understanding local adaptation requires investigating both how individual factors can act as agents of selection and how interactive effects between factors drive divergence^8^.

North-temperate zone fish species present an ideal test case for the investigation of the multifaceted nature of local adaptation to new environments. As Pleistocene glaciers receded, marine and anadromous fishes in the north-temperate zone would have been able to move into newly created freshwater habitats that provided “…diverse niche opportunities, including habitat diversity, habitat complexity, food-chain structure and other variables”^9^, and adopt a freshwater-resident life history. Although freshwater habitats presented these benefits to the new colonists, they also presented a suite of biotic and abiotic challenges such as differences in salinity, ion availability, nutrients, and temperature, which are known to influence migration patterns between the ocean and fresh water and the evolution of freshwater residency^9–18^. An interaction of particular importance for the adoption of year-round freshwater residency in the north-temperate zone is the combination of low salinity and cold winter temperatures in fresh water, which may present difficulties for fish growth and survival^19^. For anadromous fish, migration back to the sea during the winter allows them to avoid these harsh winter conditions^9,13^. As such, evolution in response to cold winter conditions and low salinity might be critical for the long-term persistence of temperate freshwater fish populations.

The threespine stickleback (*Gasterosteus aculeatus*) is a species that can provide insight into the evolution of freshwater residency. After the recession of Pleistocene glaciers in the Northern hemisphere 10,000–20,000 years ago, ancestral marine or anadromous stickleback colonised fresh water from the ocean^20–25^. This was followed by adaptation to freshwater habitats, demonstrated by the parallel evolution of morphological, physiological, and behavioural traits in freshwater populations^21,22,24–29^. Additional work has provided strong evidence that selection on growth is likely to have been present and may have played a role in the evolution of the prominent differences between marine and freshwater stickleback ecotypes^30–32^.

During their colonisation of freshwater habitats from the ocean, it is likely that stickleback faced not only the challenge of a change in salinity, but also a change in temperature. In the north-temperate zone, the temperature of freshwater habitats is more variable than the temperature of the ocean^33^, and, in particular, lakes in British Columbia become colder than the ocean in the winter^34^. Previous studies have suggested that marine stickleback are at a growth disadvantage compared to freshwater stickleback in fresh water at an intermediate temperature^30^, and that cold winter conditions reduce the growth of marine stickleback more than freshwater stickleback^35^. These two studies separately investigated different abiotic factors that may act as agents of divergent natural selection in stickleback when they evolve freshwater residency, but did not assess the interactive effects of salinity and temperature or follow the effects of these environmental differences across a winter season. Although work has investigated the interactive effects of salinity and temperature on adult stickleback survival^36^, growth has not been studied in this context. In the present study, we monitored the growth and survival of marine, anadromous, and freshwater stickleback from hatching to an age of nine months in a factorial experiment that manipulated both salinity and winter temperature in the laboratory. The factorial manipulation confers three crucial benefits over previous work: (1) the design mimics ecologically realistic conditions (e.g. cold fresh water and warm brackish water); (2) the manipulation of two factors allows for a direct comparison of their relative effects; (3) manipulating two factors in a fully factorial design allows for a test of their interactive effects. We predicted that a freshwater stickleback population would show patterns of growth consistent with local adaptation to both low salinity and cold winter temperatures when compared to patterns of growth in a marine and anadromous population.

## Methods

### Stickleback populations, acclimation conditions, and time course

Adult stickleback were collected from three populations in May and June of 2014. All collection sites were in British Columbia, and included one marine population from Oyster Lagoon (49°36′43.53″N, 124°01′52.12″W), one anadromous population from the mouth of the Little Campbell River (49°00′52″N, 122°45′33″W), and one freshwater population from Trout Lake (49°30′29″N, 123°52′29″W). Only male and female stickleback that were in breeding condition were collected, and they were used to generate progeny using the artificial fertilisation techniques outlined in ref.^30^. All experiments were conducted under University of British Columbia approved animal care protocols (#A10-0285; A11-0372).

Genetic crosses from wild-caught adults were performed at two different salinities immediately after collecting the fish: 0 ppt (dechlorinated Vancouver tap water) and 20 ppt (made using Instant Ocean ® sea salt). These salinities were chosen because they are representative of the salinities in their natal environments (Trout Lake = 0 ppt; Oyster Lagoon is approximately 20 ppt). The genetic crosses yielded the following numbers of families: 0 ppt: five families Oyster Lagoon x Oyster Lagoon (OL x OL), four families Trout Lake x Trout Lake (TL x TL), four families Little Campbell River x Little Campbell River (LC x LC); 20 ppt: six families OL x OL, four families TL x TL, six families LC x LC. Individual families were raised in 100 L glass aquaria at the University of British Columbia at the same salinity at which the genetic crosses were performed, at a water temperature of 17 °C and a photoperiod of 12 L:12D. Larval stickleback were fed live brine shrimp twice daily for the first four months, a mixture of brine shrimp and finely chopped bloodworms (Chironomid larvae) to satiation for the next three months, and roughly chopped bloodworms to satiation for the remaining two months of the experiment. Approximately one month after hatching, fish families (one family in each aquarium) were split and evenly redistributed amongst aquaria so each new aquarium contained an even mixture of fish from each individual family (from the same ecotype and salinity). From this point forward, individual families were not tracked, and thus family is not included as a unit of replication in subsequent analyses. Each aquarium had a starting fish density of 27-28 fish, with the following total numbers of aquaria: 0 ppt: eight OL x OL, four TL x TL, eight LC x LC; 20 ppt: eight OL x OL, four TL x TL, eight LC x LC. Half of the aquaria from each ecotype/salinity combination were housed in one of two environmental chambers; both chambers had an initial temperature of 17 °C and photoperiod of 12 L:12D. Five months after hatching, 10 fish from each aquarium were individually marked so that growth, in both length and mass, of individual fish could be tracked over the course of the experiment. Body length is positively correlated with egg production in female stickleback^37,38^ and is therefore a proxy for fitness in each environment.

**Figure 1:**
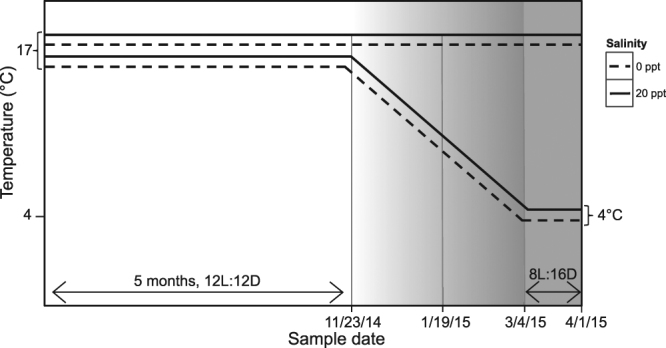
Experimental Design. All ecotypes (anadromous (Little Campbell River), marine (Oyster Lagoon), freshwater (Trout Lake)) were hatched and raised in salinities of 0 ppt and 20 ppt for 5 months at 12 L:12D photoperiod. After 5 months, starting on 11/23/14, the light period was decreased at a rate of 20 minutes per week and reached a minimum of 8 L:16D (solid grey shading) at 2/24/15 for all groups. Over the same period, the temperature in the experimental tanks was decreased by 0.13 °C per day, reaching a minimum of 4 °C on 3/4/15, while the control tanks were maintained at 17 °C.

Five months after hatching, the photoperiod in both chambers was switched to mimic that of the natural environment (photoperiod was gradually transitioned from 12 L:12D to 8 L:16D by reducing the amount of light by 20 minutes per week). Photoperiod was altered to simulate seasonal conditions during fall and winter, as changes in photoperiod that precede winter are a critical cue for changes in physiology and energy metabolism that are important for winter survival and preparation for reproduction^39–42^. Temperature was maintained at 17 °C for the entirety of the experiment in the ‘control’ environmental chamber, but five months after hatching, temperature was gradually decreased by 0.13 °C per day to reach a minimum of 4 °C in the ‘experimental’ environmental chamber (Fig. 1). 17 °C and 4 °C were chosen because 17 °C is representative of the monthly mean temperature during the summer months and 4 °C is representative of the monthly mean temperature during the winter months in freshwater lakes in British Columbia^34^. Note that because there was only one environmental chamber designated as the control environment and one environmental chamber designated as the treatment environment, temperature and chamber effects are confounded.

Fish length and mass were measured on all fish in each aquarium at four points in both environments (Fig. 1): 1) Five months (151 days) post-hatching, immediately before temperature reduction; 2) 57 days after the start of temperature reduction at which point the temperature in the experimental condition had reached 9.5 °C; 3) at the end of the temperature reduction phase (44 days after the second sampling); 4) 28 days after reaching the minimum temperature of 4 °C.

### Analysis of growth data and growth in relation to degree-days

To investigate the effects of salinity, temperature, and stickleback ecotype on mass and length, we carried out linear mixed effects models (LME)^43^ at the time of first sampling and the final sampling, implemented in R. LME models for mass and length at first sampling included salinity and stickleback ecotype as fixed effects and rearing tank as a random effect to explicitly investigate the influence of salinity on early growth of each ecotype. To assess the overall effects of salinity, temperature, and ecotype we included all three variables as fixed effects in LME models with final mass and length data, and we also included rearing tank as a random effect. These LME models were used to assess stickleback size both at the early and late growth stages of the study.

To test explicitly for the effects of experimental treatments on growth rate, we calculated specific growth rate (SGR; for both mass and length) as the difference in log-transformed mass (and length) between sample periods, divided by the time of growth in days. We used LME models with salinity, temperature, stickleback ecotype, and sample period as fixed effects and individual and rearing tank as random effects, to determine the effects of experimental treatments over time on SGR. We examined each response variable for normality^44^ and transformed the data if necessary before carrying out statistical tests. Individually marked fish within each tank were the unit of replication for mass and length data.

To assess stickleback growth relative to temperature conditions, stickleback growth was standardised to degree-days (DD). Degree-days are a measure of the amount of ambient thermal energy that an ectotherm experiences^45^, and thus expressing growth relative to degree-days allows for the explicit investigation of growth independent of ambient thermal energy. We calculated the number of degree-days experienced by the control and experimental treatments between the last two sampling periods when the temperatures were the most divergent. Degree-days were calculated using the following formula:

$${\rm{D}}{\rm{D}}=\sum _{d=0}^{n}{T}_{d}-{T}_{o}$$

where DD = cumulative degree-days, T_*d*_ is the temperature on a given day, and T_*o*_ is the base temperature, which is the temperature at which growth no longer occurs^45^. Previous studies estimated values of T_*o*_ in stickleback as 3 °C^46^ and 3.5 °C^47^. As such, and based on the results observed in the current study, we used a value of 3 °C for T_*o*_. We employed an LME model with salinity, temperature, and stickleback ecotype as fixed effects and used rearing tank as a random effect.

### Mortality

We also tested for the effects of experimental treatments on mortality. We used a generalised linear mixed model approach implemented in the ‘lme4’ package in R^48^ with salinity, temperature, and stickleback ecotype as fixed effects to understand how these treatments, and the interactions between them, altered total mortality. Mortality data were counts and showed a pattern of overdispersion, so we specified a quasi-Poisson distribution, which includes a term that fits overdispersion, for the distribution in the model^49^.

All LME models in the study were analysed using a Wald chi-square test to calculate chi-square and p-values for each LME^50^ (‘Anova’ command, ‘car’ package in R^51^).

### Data Availability

All data generated or analysed during this study are included in this published article (and its Supplementary Information files).

## Results

### Mass and length at the first sampling point

**Figure 2:**
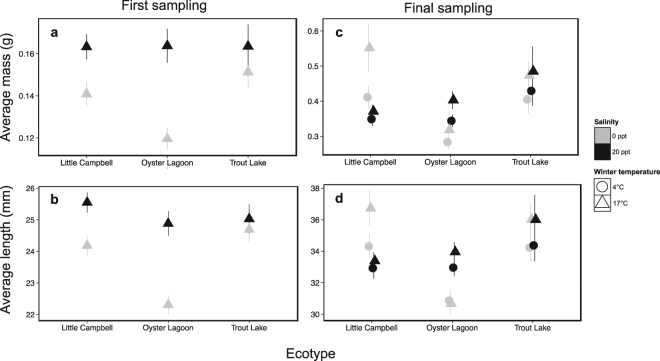
Mean mass and length of stickleback at the first and final sampling periods. Panel a, b: mass and length, respectively, of stickleback at the first sampling period. Panel c, d: mass and length, respectively, of stickleback at the final sampling period. Little Campbell River = anadromous ecotype; Oyster Lagoon = marine ecotype; Trout Lake = freshwater ecotype.

During the first phase of the experiment, the stickleback ecotypes were reared at different salinities, but at a common temperature of 17 °C. At this initial sampling point we observed a direct effect of salinity (df = 1,28, Chisq = 12.92, p = 0.0003) on mass (Fig. 2a), with fish being larger at higher salinities. Similarly, we observed a significant effect of salinity (df = 1,34, Chisq = 11.40, p = 0.0007) on length (Fig. 2b), but in this case we also observed a significant effect of ecotype (df = 1,34, Chisq = 3.25, p = 0.039) with no significant interaction. Although we did not detect a significant interaction between ecotype and salinity for either mass or length, in general the largest effect of salinity was observed for the marine ecotype (where at lower salinities marine fish tended to be smaller), and the smallest effect of salinity was observed for the freshwater ecotype. Mass and length across time during the experiment are presented in Supplementary Fig. S1.

### Mass and length at the last sampling point

By the end of the experiment fish had been exposed to both different temperatures and different salinities in a fully factorial design. At the final sampling point we observed significant effects of ecotype (df = 2,22, Chisq = 7.95, p = 0.019), temperature (df = 1,22, Chisq = 4.24, p = 0.040), and the interaction between salinity and ecotype (df = 2,22, Chisq = 6.54, p = 0.038) on final mass (Fig. 2c). Similarly, for final length, we uncovered both significant effects of ecotype (df = 2,22, Chisq = 8.70, p = 0.0123) and an interaction between salinity and ecotype (df = 2,22, Chisq = 7.18, p = 0.028) (Fig. 2d). These significant interactions suggest that the ecotypes differ in their response to salinity.

### Specific growth rate

**Figure 3:**
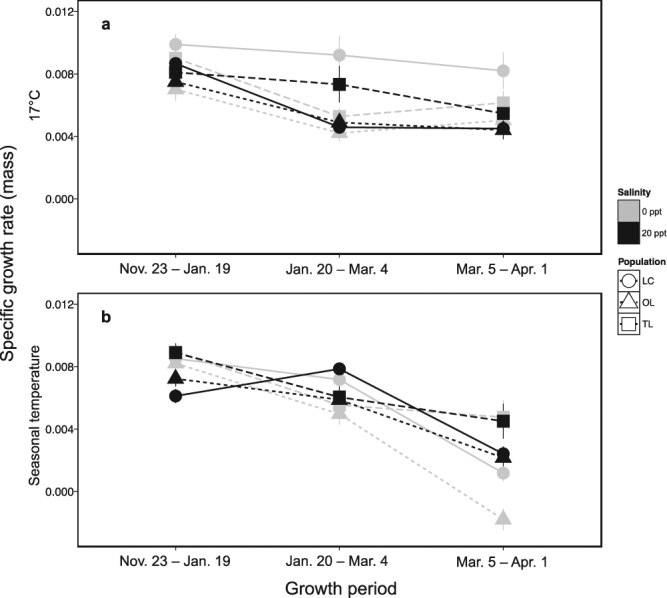
Specific growth rates (SGR) for mass between sampling points. Panel a: stickleback held at 17 °C for the duration of the study. Panel b: stickleback that experienced declining temperatures. All data are expressed as mean ± SEM. (See Supplementary Fig. S2 for SGR for length). LC = Little Campbell River (anadromous ecotype); OL = Oyster Lagoon (marine ecotype); TL = Trout Lake (freshwater ecotype).

Figure 3 presents the effects of temperature, salinity, and ecotype on specific growth rate (SGR) for mass over time. SGR for mass was significantly affected by ecotype (df = 2,22, Chisq = 12.80, p = 0.0017) and temperature (df = 1,22, Chisq = 7.39, p = 0.006). In addition, there was an ecotype by time interaction (df = 4,424, Chisq = 10.02, p = 0.04), with marine and anadromous fish showing a steeper decline in SGR over the course of the study. Furthermore, there was a temperature by time interaction (df = 2,424, Chisq = 60.30, p < 0.0001), with SGR decreasing more slowly in the 17 °C control conditions than in the experimental conditions. There was also a salinity by time interaction (df = 2,424, Chisq = 7.23, p = 0.027), with fish showing a higher SGR in 20 ppt early in the first two growth periods. We also observed a salinity by temperature interaction (df = 1,22, Chisq = 3.97, p = 0.047), with fish kept at 17 °C having a higher SGR when exposed to 0 ppt in contrast to fish kept at 4 °C having a higher SGR when kept at 20 ppt. Furthermore, there was a temperature by ecotype by time interaction (df = 4,424, Chisq = 22.66, p = 0.0001), driven by rapidly declining SGR of both marine and anadromous fish when they were kept in cold experimental winter conditions. We also observed a temperature by salinity by time interaction (df = 2,424, Chisq = 15.18, p = 0.0005) that was driven by rapidly declining SGR when fish experienced 4 °C experimental winter conditions and 0 ppt. Finally, we found an ecotype by salinity by temperature by time interaction (df = 2,424, Chisq = 22.40, p = 0.022) that stemmed from differences in SGR between marine and freshwater fish increasing over time when held in the 4 °C and 0 ppt experimental winter conditions. Overall, these data suggest that winter cold has a strong negative effect on the growth of both marine and anadromous fish regardless of salinity, and that these effects are even greater in marine fish held in fresh water.

### Growth in relation to degree-days

**Figure 4:**
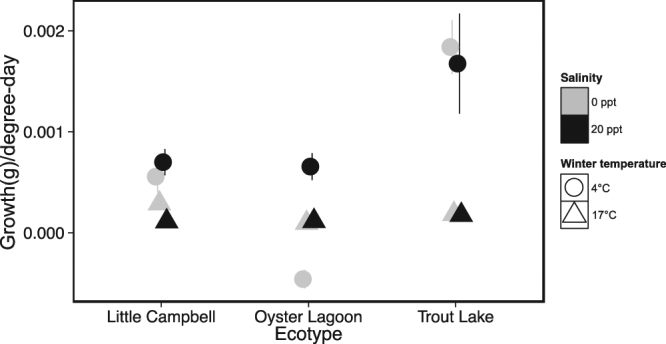
Growth per degree-day during the final sampling period (3/5/15-4/1/15). All data are expressed as mean ± SEM. Little Campbell River = anadromous ecotype; Oyster Lagoon = marine ecotype; Trout Lake = freshwater ecotype.

Because of thermodynamic effects on metabolism, low temperature is expected to have a strong negative effect on growth rate in ectotherms. In principle, these thermodynamic effects can be accounted for by normalising growth rate to the number of degree-days (DD) experienced. In this experiment, the number of degree-days was the most different between temperature treatments during the last growth period (between sample points 3 and 4; experimental conditions = 29 DD; control conditions = 406 DD). Figure 4 displays growth rate normalised to degree-days for this final sampling period. There was an effect of ecotype (df = 2,22, Chisq = 19.73, p < 0.0001) and winter temperature (df = 1,22 Chisq = 26.15, p < 0.0001) on growth expressed relative to degree-days. In addition, there was a significant interaction between ecotype and winter temperature (df = 2,22, F = 18.46, p < 0.0001), with the freshwater ecotype showing a much greater ability to grow in cold conditions than the anadromous or marine ecotypes.

### Mortality

**Figure 5:**
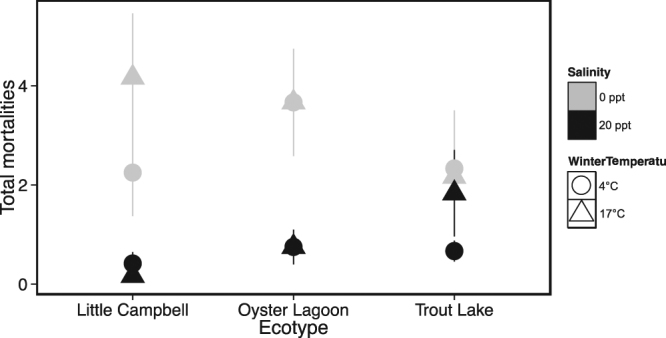
Mortalities over the course of the experiment. Data are presented as the mean of the number of mortalities per tank ± SEM. Little Campbell River = anadromous ecotype; Oyster Lagoon = marine ecotype; Trout Lake = freshwater ecotype.

There were 2,782 total fish in the experiment and 232 total mortalities during the study. Salinity had an effect on mortality (df = 1, Chisq = 40.50, p < 0.0001), as tanks with a salinity of 0 ppt had an average of 3.20 mortalities compared to 0.50 mortalities for tanks with a salinity of 20 ppt (Fig. 5). In addition, there was a non-significant trend toward an interactive effect of salinity and ecotype on mortality (df = 2, Chisqi = 5.15, p = 0.064), with tanks of marine fish kept at 0 ppt having an average of 3.67 mortalities compared to 0.21 mortalities on average for anadromous fish kept at 20 ppt.

## Discussion

When colonising novel environments, populations may immediately face changes in a number of abiotic conditions, each of which may impose selection. In addition, combinations of novel abiotic factors may pose particularly strong barriers to residence in novel environments. Disentangling the effects of each abiotic difference and their interactions is a crucial step in identifying the abiotic challenges associated with the colonisation of novel environments. In the north-temperate zone, both low salinity and low winter temperatures have the potential to act as abiotic factors that could pose a barrier to permanent colonisation of fresh water, but few studies have evaluated the relative roles of these factors during colonisation and during subsequent evolutionary change. Ancestral anadromous stickleback leave fresh water to migrate to the ocean prior to the onset of cold winter temperatures^52^, and thus do not naturally experience the combination of low temperature and low salinity that is characteristic of freshwater habitats in the north-temperate zone. Here we show that both salinity and temperature, and the interaction between these abiotic factors, differentially alter the growth and size of stickleback ecotypes with different evolutionary histories. We assessed growth in only one population of each ecotype, limiting our power to draw conclusions about differences between freshwater, anadromous, and marine populations generally. However, despite this limitation, our data suggest that growing in low salinity conditions is a challenge for marine and anadromous fish. Temperature also caused profound differences in growth between ecotypes, as marine fish showed a substantial reduction in growth at low temperatures, while freshwater stickleback were able to maintain relatively high growth rates at the coldest winter temperatures. The striking ability of freshwater stickleback to maintain growth rates in cold fresh water, in concert with previous work on differences in thermal tolerance among the ecotypes^34,35^, suggests that cold overwintering temperatures may be a key agent of natural selection for marine stickleback colonising temperate freshwater environments, and provides insight into the factors driving adaptation during colonisation of freshwater habitats.

We observed an interaction between salinity and ecotype on both mass and length in stickleback at later life stages. This interactive effect likely stemmed from faster growth over time by freshwater stickleback in 0 ppt conditions. For example, at the time of first sampling, freshwater stickleback were 20% heavier than marine stickleback in fresh water (Fig. 2a), but by the end of the study this difference had increased to 33% (Fig. 2c). Low salinity also had a negative effect on survival, with both the marine and anadromous ecotypes having much higher mortality at low salinity than at high salinity (Fig. 5). The superior growth of the freshwater ecotype relative to the marine ecotype prior to temperature reduction (Fig. 2b) may be beneficial by allowing higher initial levels of pre-winter energy storage^42^. This is critical because smaller fish have higher mass-specific metabolic rates and lower lipid storage capacity, contributing to a high risk of winter mortality^42,53,54^. Therefore, freshwater stickleback may have an advantage during overwintering in fresh water due to a superior ability to store energy prior to winter and deplete these energy stores more slowly than marine and anadromous ecotypes^42^.

One possible mechanism underlying the negative effects of low salinity on the growth of the fully plated marine ecotype could be the low concentrations of calcium associated with fresh water, as freshwater colonisation is associated with reductions in bony structures such as lateral plates, spines, and the pelvic girdle in stickleback^55–58^. For example, the growth of completely plated stickleback is inhibited to a greater degree than the growth of low plated stickleback at low calcium concentrations, providing evidence that low calcium concentration may have been an important selective agent driving major differences between marine and freshwater ecotypes^57^. Furthermore, stickleback ecotypes may differ in their ability to take up calcium from their environment. In dilute fresh water, where stickleback are confronted with the problem of diffusive ion loss, the epithelial calcium channel (ECaC) is an ion transporter located in the fish gill that is utilised to actively pump calcium from dilute fresh water back into the blood plasma^59,60^. We have previously shown that freshwater stickleback exhibit higher gene expression of ECaC than marine stickleback in fresh water, independent of decreases in temperature and photoperiod associated with winter conditions^35^. Increased ECaC expression in the freshwater ecotype has the potential to play a beneficial role in calcium uptake and the improved growth of freshwater stickleback in fresh water. Alternatively, other physiological mechanisms that control growth could differ among stickleback populations. For example, previous work has detected differences in the thyroid hormone axis between stickleback ecotypes^1^, and such differences could also play an important role in determining the differences in growth that we observed. In addition, there is evidence of divergence in the insulin-like growth factor pathway between stickleback ecotypes^61–63^, and this pathway is known to be important for determining growth in fish^64^. Taken together, these data highlight several potential mechanisms that could underlie the growth differences that we observed.

During the last sampling period in our experiment, when temperatures were the most divergent between the two treatment groups (Fig. 1), we observed striking differences in growth between ecotypes. Specific growth rate (SGR) (Fig. 3) showed an ecotype, by temperature, by salinity, by time interaction, suggesting that the interaction between ecotype, winter temperature, and salinity plays a major role in dictating daily growth of stickleback at low winter temperatures. Because of the reduced thermal energy in the environment at low temperatures, the growth rate and metabolic rate of ectotherms are expected to decline in the cold, and this effect is evident when examining SGR for both mass and length during the final sampling period (Fig. 3 and Supplementary Fig. S2, respectively). However, these effects of low temperature differed among ecotypes. Freshwater stickleback showed only a small (~20%) reduction in SGR for mass when experiencing 4 °C winter conditions (i.e. between the third and final sampling period), while marine and anadromous stickleback showed sharp declines (82% and 74% respectively) under these same 4 °C winter conditions (Fig. 3b). This difference cannot be explained by inherent differences in growth rate among the ecotypes, as freshwater, marine, and anadromous stickleback showed similar small declines in growth rate during this same life stage when kept at 17 °C (8%, 2%, and 5% respectively) (Fig. 3a). The relatively small reduction in SGR in the freshwater ecotype in the cold is unexpected, because in the absence of physiological compensation, rate processes such as growth would be expected to decline by ~2-3 fold (50–66%) for each 10 °C decrease in temperature. Our data strongly suggest that freshwater stickleback are able to induce physiological compensatory mechanisms to reduce or prevent the expected thermodynamically-driven reduction in growth, and that these mechanisms are not available, or not as effective, in marine or anadromous stickleback. Previous work has suggested differential gene expression plasticity in response to temperature among stickleback ecotypes^65^, and these or other differences in gene expression plasticity have the potential to play a role in shaping the growth differences we observe here.

The population-level differences in growth in cold conditions were magnified in fresh water. Freshwater stickleback showed similar growth regardless of salinity in the cold, anadromous fish had somewhat reduced growth in cold fresh water compared to cold 20 ppt water, whereas marine fish were only able to maintain growth in cold water at 20 ppt and showed a negative growth rate in cold fresh water. It is possible that freshwater stickleback may have less permeable gills than marine stickleback in fresh water^62^, potentially resulting in higher energetic costs associated with ion uptake for marine stickleback in fresh water. These higher costs could result in lower growth rates for marine stickleback in fresh water. As ion regulation is known to be particularly challenging in the cold^66^, this presents a potential mechanism explaining the poor growth of marine fish in cold fresh water.

The differences in growth between ecotypes at low temperature and low salinity are most evident when growth is examined in terms of “degree-days” (Fig. 4). Degree-days represent the cumulative thermal energy available for growth^45^. During the last sampling period growth per degree-day was very similar among the ecotypes and was not affected by salinity when fish were held at 17 °C (Fig. 4). In contrast, when fish were exposed to low temperature, the freshwater ecotype had much higher growth per degree-day than did the other two ecotypes, clearly demonstrating that this ecotype has superior ability to grow in the cold. In addition, the growth of the marine ecotype was severely affected by the combination of cold temperature and low salinity, whereas the growth of the other two ecotypes was not negatively affected by low salinity at either temperature (Fig. 4). These divergent patterns among the ecotypes strongly suggest that overwintering in fresh water may have presented a challenge to freshwater colonisation for stickleback in the north-temperate zone.

Our results are consistent with previous work that has suggested that the combination of cold and fresh water may have presented a challenge to freshwater colonisation in stickleback. For example, freshwater stickleback have evolved superior acute cold tolerance relative to that of the ancestral marine form^34,35^, suggesting that low temperatures act as a selective force driving divergence in stickleback. However, differences in cold tolerance between ecotypes are abolished after acclimation to winter conditions^35^, indicating that poor tolerance of acute exposure to cold temperatures cannot be a major barrier to freshwater colonisation. The data presented here suggest that cold temperatures drive differences in growth rate between marine and freshwater ecotypes during the winter in fresh water, which represents a potential fitness cost during freshwater colonisation. Although we have examined only single marine, anadromous, and freshwater populations in this study, our results suggest that these freshwater fish may have adapted to growing at cold winter temperatures likely only experienced in fresh water, which could represent a case of fairly rapid adaptation in a key physiological process in response to a challenging thermal regime. Future work to determine whether freshwater colonisation by marine stickleback in the north-temperate zone often includes adaptation to the dual conditions of low salinity and low temperature are crucial to understanding the generality of these findings. Of particular interest would be future work comparing growth of marine and freshwater threespine stickleback populations in regions of cold fresh water across the species range.

As a whole, our study demonstrates that these two abiotic factors, and the interaction between them, can shape the performance of closely related populations. In addition to temperature and salinity, previous work has identified nutrient availability^67^ as a potential axis of variation, and many more factors could also be important. As such, investigations into what drives physiological adaptation should most often include a manipulation of multiple factors; simply because environments often differ along a number of axes. This view may seem daunting, as experiments quickly become less tractable as more treatments are added, but in many cases it adds crucial realism to the investigation of physiological adaptation.

## Electronic supplementary material

Supplementary Information
