# Supplementary material for: Low temperature and low salinity drive putatively adaptive growth differences in populations of threespine stickleback
Source: Sci Rep. 2017 Dec 1;7:16766. doi: 10.1038/s41598-017-16919-9 (PMC5711929; doi:10.1038/s41598-017-16919-9)
Supplement: Supplementary file 1 — Supplementary Information [file 41598_2017_16919_MOESM1_ESM.pdf]

Supplementary Information for:

Low temperature and low salinity drive putatively adaptive growth differences in populations of threespine stickleback

Taylor C. Gibbons*, Seth M. Rudman, and Patricia M. Schulte

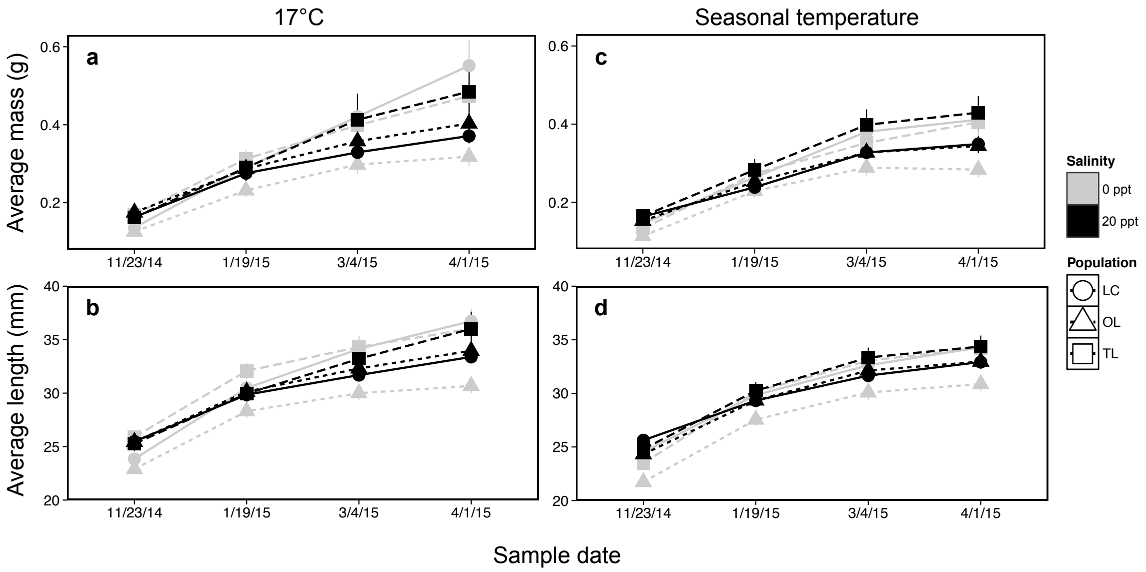

Figure S1. Mean mass and length of stickleback during the course of the study. Panel a, b: mass and length, respectively, of stickleback held at 17°C for the duration of the study. Panel c, d: mass and length, respectively, of stickleback that experienced declining temperatures. Note: the x-axis is not to scale and connecting lines are provided for visual clarity only. LC = Little Campbell River (anadromous ecotype); OL = Oyster Lagoon (marine ecotype); TL = Trout Lake (freshwater ecotype). All data are presented as mean ± SEM.

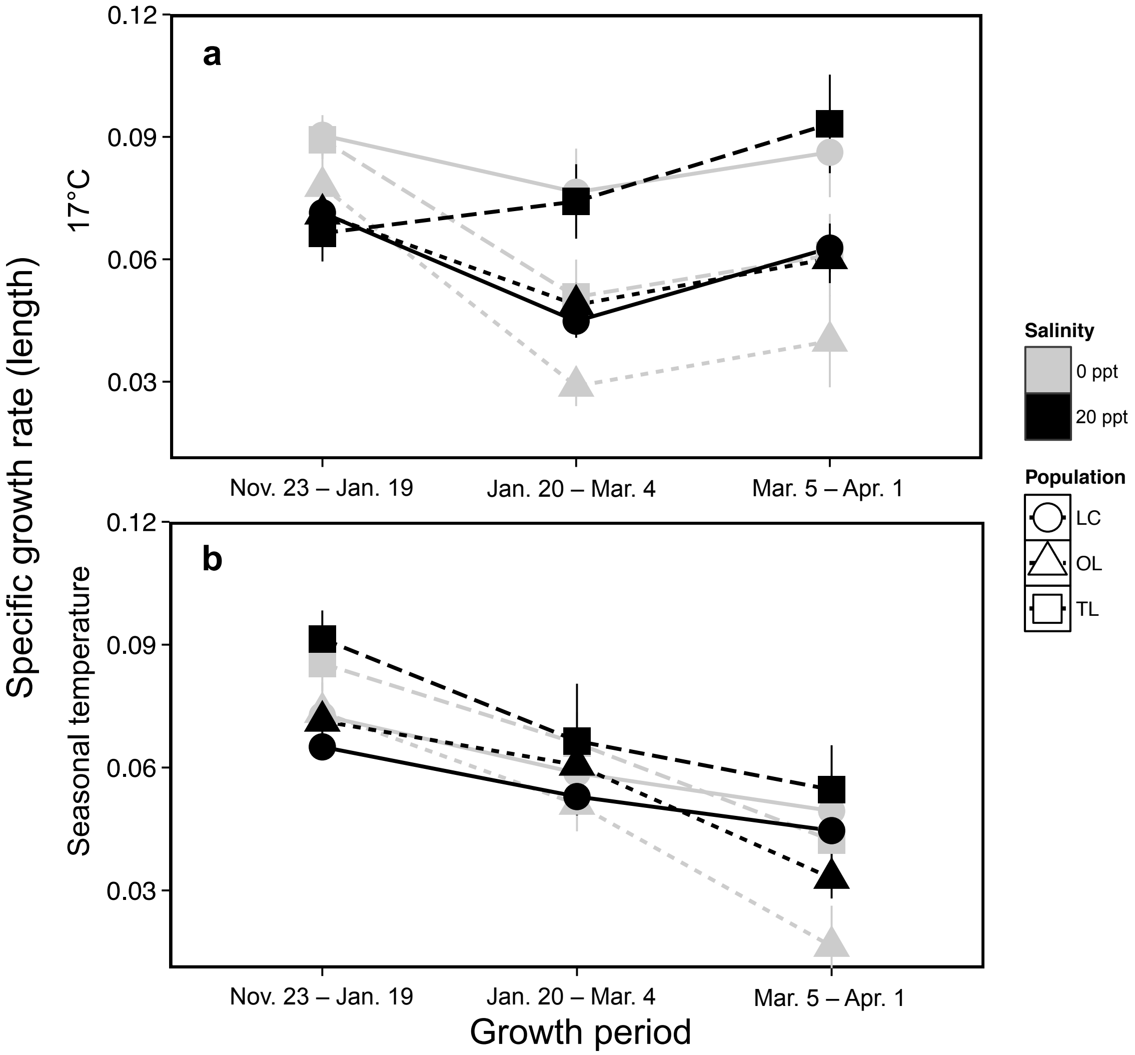

Figure S2. Specific growth rates (SGR) for length between sampling points. Panel a: stickleback held at 17°C for the duration of the study. Panel b: stickleback that experienced declining temperatures. All data are expressed as mean ± SEM. LC = Little Campbell River (anadromous ecotype); OL = Oyster Lagoon (marine ecotype); TL = Trout Lake (freshwater ecotype).
